# Divergent and overlapping roles of homospermidine and spermidine in Sinorhizobium meliloti physiology and symbiotic performance

**DOI:** 10.1099/mic.0.001668

**Published:** 2026-02-06

**Authors:** Víctor A. Becerra-Rivera, Alejandra Arteaga Ide, Alma R. Reyes-González, Michael F. Dunn

**Affiliations:** 1 Programa de Genómica Funcional de Procariotes, Centro de Ciencias Genómicas, Universidad Nacional Autónoma de México, Cuernavaca, Morelos, C. P. 62210, Mexico; 2 Universidad Nacional Autónoma de México, Cuernavaca, C. P. 62210, Morelos, Mexico

**Keywords:** biofilms, carboxyspermidine dehydrogenase, homospermidine synthase, motility, polyamine biosynthesis, *S. meliloti*-alfalfa symbiosis

## Abstract

Unlike most rhizobia, *Sinorhizobium meliloti* produces spermidine (Spd) in addition to putrescine (Put) and homospermidine (HSpd) as soluble intracellular polyamines. To investigate their roles, we analysed *S. meliloti* Rm8530 mutants lacking *hss* (homospermidine synthase, *smc04016*) or *casdh* (carboxyspermidine dehydrogenase, *smb21630*), as well as a double mutant. Biochemical and phenotypic characterization confirmed that *hss* and *casdh* are responsible for HSpd and Spd synthesis, respectively, and showed that these structurally similar molecules exert both distinct and overlapping physiological functions. The *hss* and *hss casdh* mutants exhibited reduced swimming motility, which was fully restored by HSpd or *hss* complementation, but not by Spd or *casdh*. In contrast, swarming motility defects in the double mutant were rescued by either gene or polyamine. Biofilm formation and exopolysaccharide production were largely unaffected. The *hss* mutant grew normally in minimal medium and formed effective symbioses with alfalfa, whereas the *casdh* mutant showed slightly delayed growth and reduced nitrogen fixation. The double mutant displayed a pronounced growth lag and significantly lower plant biomass and nitrogen fixation. The expression of *hss* and *casdh* was lower in the quorum-sensing–competent strain Rm8530 than in the quorum sensing–deficient strain 1021, with *hss* expressed about tenfold higher than *casdh* despite Spd being more abundant in the cells. These results highlight complementary and partially interchangeable roles of spermidine and homospermidine across *S. meliloti* growth and symbiotic functions.

## Introduction

Rhizobia are free-living bacteria in soil and rhizospheres that can enter into nitrogen-fixing endosymbioses with legumes. They play a vital role in the global nitrogen cycle and are essential contributors to sustainable agriculture by providing a natural source of bioavailable nitrogen for plants.

Polyamines are aliphatic hydrocarbons containing two or more amino groups that act as intracellular physiological modulators [1]. In many bacteria, endogenously synthesized polyamines or those taken up from the environment markedly influence a variety of physiological processes in free life as well as interactions with eukaryotic hosts, including those between rhizobia and legumes [1,4]. In the alphaproteobacterium *Sinorhizobium meliloti*, endogenous or exogenous polyamines affect exopolysaccharide (EPS) production, motility, growth, abiotic stress resistance, biofilm formation and symbiotic efficiency with alfalfa (*Medicago sativa*) [3,5, 6].

**Fig. 1.**
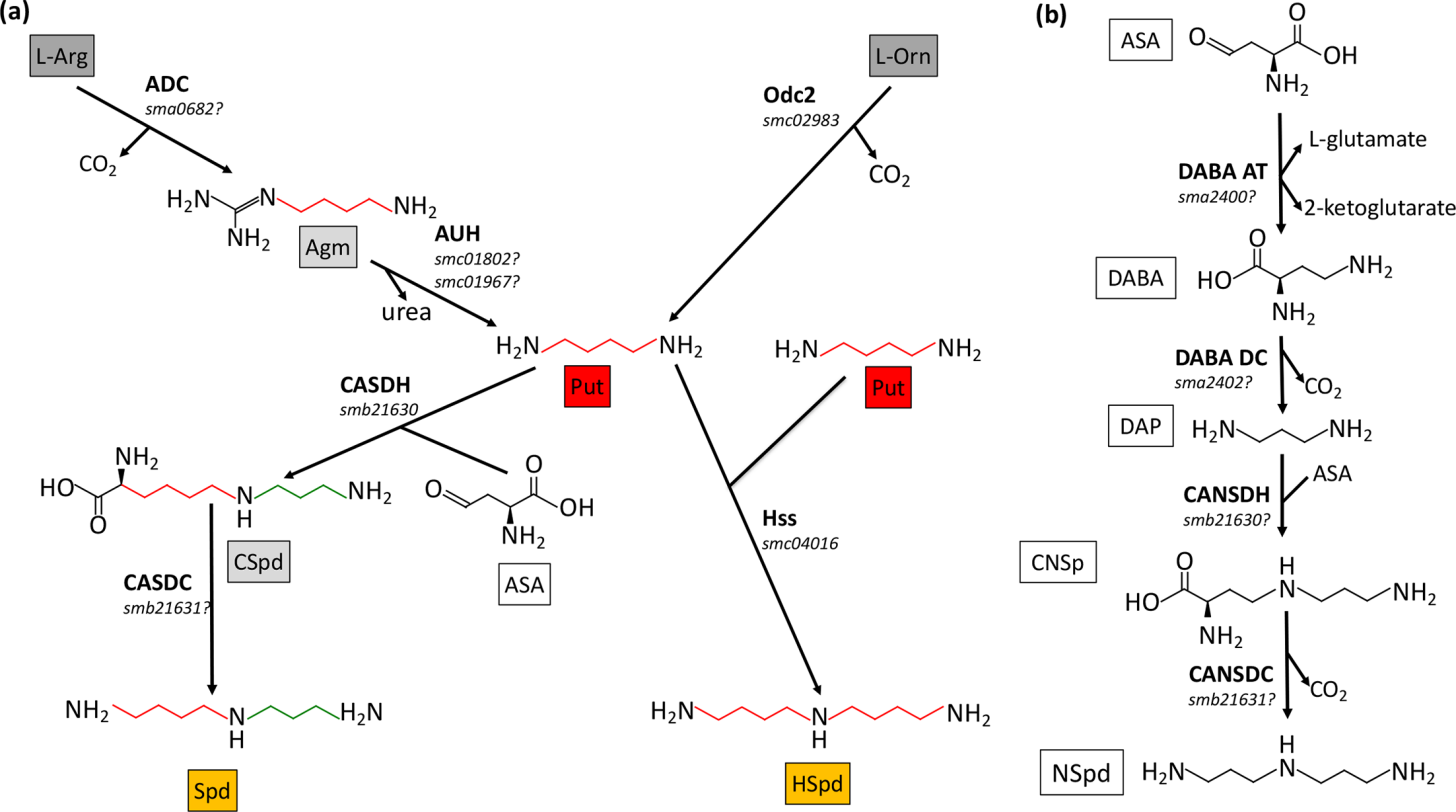
Polyamine biosynthesis in *S. meliloti*. (**a**) Enzymes and their encoding genes in the pathways for putrescine (Put), spermidine (Spd) and homospermidine (HSpd) in *S. meliloti*. Ornithine decarboxylase 2 (Odc2) activity is the major source of Put, while the predicted participation of Hss and Casdh in HSpd and Spd synthesis [5] was verified in the present work. (**b**) The possible NSpd biosynthesis pathway. For both panels, question marks identify genes encoding products whose participation in the pathways has not been experimentally verified. Abbreviations not defined in the text: Agm, agmatine; l-Arg, l-arginine; ASA, aspartate-*β*-semialdehyde; AUH, agmatine ureohydrolase (agmatinase); CSpd, carboxyspermidine; DABA, diaminobutyric acid; DABA AT, DABA aminotransferase; DABA DC, DABA decarboxylase; DAP, 1,3-diaminopropane; l-Orn, l-ornithine.

Most symbiotic nitrogen-fixing rhizobia produce the diamine putrescine (Put) and the triamine homospermidine (HSpd) as their only soluble cytoplasmic polyamines, while *S. meliloti* is also able to produce the triamines spermidine (Spd) and norspermidine (NSpd) (Fig. 1a, b) [2,5, 7]. We have shown that virtually all of the Put produced by *S. meliloti* Rm8530 is synthesized by ornithine decarboxylase 2 (Odc2; Fig. 1a) [5]. Based on genome analysis, we predicted that HSpd is produced by the condensation of two molecules of Put by the homospermidine synthase (Hss) encoded by locus *smc04016*. We also proposed that Spd is synthesized in two steps from Put by the enzymes carboxyspermidine dehydrogenase (Casdh) (annotated as a saccharopine dehydrogenase family protein, SMb21630) and carboxyspermidine decarboxylase (Casdc; SMb21631) [5,7] (Fig. 1a). Casdh and Casdc may also participate in the synthesis of NSpd (Fig. 1b), previously identified in the insoluble polyamine fraction in *S. meliloti* Rm8530. In contrast to Spd and HSpd, NSpd does not use Put as a precursor [5] (Fig. 1b).

Spd and HSpd have been shown to impact the physiology of several species of rhizobia. HSpd synthesis by *Rhizobium tropici* contributed to this organism’s resistance to salt stress during symbiosis with bean plants [8]. A *Rhizobium etli hss* null mutant was incapable of swarming motility and had a slightly slower growth rate in rich medium but was not affected in swimming motility *in vitro* or nitrogen fixation with bean plants [9]. In the non-nitrogen-fixing rhizobial species *Agrobacterium tumefaciens*, Spd is required for growth and modulates biofilm formation, while HSpd does not appear to have a role in either process [10].

We reported previously that biofilm formation, motility, EPS production and symbiotic efficiency were altered in a *S. meliloti* Rm8530 *odc2* mutant, which produces very low levels of Put, Spd and HSpd. Because the *odc2* mutant is deficient in synthesizing three polyamines, we could not unambiguously attribute specific phenotypic effects to individual polyamines [5,6]. In some cases, phenotypic alterations occurring in bacterial polyamine biosynthesis mutants can be reversed by supplying a different polyamine to the mutant, while in others, the functions of specific polyamines cannot be compensated for [1,4, 10]. 

The physiological roles of Spd and HSpd have not been specifically addressed in *S. meliloti*. The purpose of this work was to confirm the functionality of the *hss* and *casdh* genes and to use *S. meliloti* null mutants in either or both genes to determine if these chemically similar polyamines (Fig. 1) have distinct or functionally redundant roles in growth, motility, biofilm formation and symbiosis with alfalfa. The results presented here show that certain phenotypes depend specifically on the ability to synthesize either Spd or HSpd, whereas other mutant defects can be complemented by either polyamine.

## Methods

### Bacterial strains, plasmids and culture media

Bacterial strains and plasmids are listed in Table 1. Peptone-yeast extract (PY) and Luria broth complex media and minimal medium succinate ammonium (MMSA) were prepared as described previously [5]. Antibiotics were used at final concentrations (μg ml^−1^) of the following: carbenicillin (Cb), 50; gentamicin (Gm), 15; kanamycin (Km), 50; spectinomycin (Sp), 100; and streptomycin (Sm), 200. Polyamine supplements were prepared as concentrated stocks, adjusted to pH 6.8 and filter sterilized. Standard protocols were used to grow *Escherichia coli* [11].

**Table 1.** Strains and plasmids used in this study

Strain or plasmid	Relevant characteristic	Source or reference
***E. coli* strains**		
DH5*α*	Cloning strain	Laboratory collection
Mach 1	Cloning strain	Invitrogen
***S. meliloti* strains**		
1021	Sm^r^ derivative of wild-type strain SU47, Sm^r^	[31]
Rm8530	*S. meliloti* 1021 *expR*^+^, Sm^r^	[13,14]
8530-casdh	*S. meliloti* Rm8530 *smb21630*::ΩSp *casdh* null mutant, Sm^r^ Sp^r^	This study
8530-hss	*S. meliloti* Rm8530 *smc04016*::*loxP* Sp *hss* null mutant, Sm^r^ Sp^r^	This study
Hss(8530)loxP CRE43	8530-hss containing plasmid pBBRMCre for the removal of the loxP Sp interposon from *hss*	This study
8530-hss-casdh	*S. meliloti* Rm8530 *hss casdh* double mutant	This study
**Plasmids**		
pBB5	Broad-host-range vector pBBR1MCS-5, Gm^r^	[32]
pBB5-hss	pBBR1MCS-5 containing *smc04016* with native promoter and terminator regions, Gm^r^	This study
pBB5-casdh	pBBR1MCS-5 containing *smb21630* with native promoter and terminator regions, Gm^r^	This study
pBB53	∆*placZ* pBBR1MCS-5 derivative with promoterless *gusA*, Gm^r^	[33]
pBB53hss::gusA	Transcriptional *smc04016*::*gusA* fusion in pBB53	This study
pBB53casdh::gusA	Transcriptional *smb21630*::*gusA* fusion in pBB53	This study
pTZ-hss	pTZ57R/T containing a 2.5 kb fragment including *hss* and flanking regions, Cb^r^	This study
pTopo-casdh	pCR2.1Topo containing a 2.3 kb fragment including *casdh* and flanking regions, Km^r^	This study
pTZ57R/T	InsTAclone vector for cloning PCR products, Cb^r^	Thermo
pTZ-hss::loxSp	pTZ57R/T with loxP Sp inserted into the *Sal*I site of the *hss* gene, Sp^r^ Cb^r^	This study
pJQ200SK+	Suicide vector for gene replacement, Gm^r^	[34]
pJQ-hss::loxSp	loxP Sp interrupted *hss* gene cloned in pJQ200SK^+^	This study
pJQ-casdh::ΩSp	ΩSp interrupted *casdh* gene cloned in pJQ200SK+	This study
pHP45ΩSp	Source of the ΩSp element, Sp^r^	[35]
pK18mobsacB	Broad-host range gene replacement vector, Km^r^	[36]
pMS102loxSp17	Source of the loxP Sp interposon, Sp^r^	[37]
pRK2013	Helper plasmid, Km^r^	[38]
pCR2.1Topo	Vector for cloning PCR products, Km^r^	Invitrogen
pBBRMCre	Plasmid used for deleting the loxP Sp interposon inserted in *smc04016, hss*	[39]

### Bacterial growth and polyamine analysis

*S. meliloti* strains were pre-cultured in PY medium, washed and inoculated into MMSA containing 1 µg ml^−1^ d-biotin and one-half the normal concentration of selective antibiotics as described in [5]. After 24 h, cells from these cultures were washed twice in MMSA containing biotin and used to inoculate a second subculture of fresh MMSA plus biotin without antibiotics. The cultures were incubated at 200 r.p.m., 29 °C and growth monitored by OD at 620 nm [5]. To quantitate polyamines, cells were obtained from 24 h MMSA cultures, and free polyamines were extracted and derivatized with dansyl chloride. These samples were analysed by high performance TLC (HPTLC) and spot intensities quantitated using the ImageJ program as described previously [5]. For use as standards, Put dihydrochloride, cadaverine, Spm, Spd, 1,3-diaminopropane and NSpd were purchased from Merck (Darmstadt, Germany) and HSpd trihydrochloride was obtained from Santa Cruz Biotechnology (Dallas, TX, USA). Three-hundred nanograms per microlitre solutions of the polyamines (as the free base, correcting for the mass of the hydrochloride where required) were prepared in 5% TCA, and 40 µl of a mixture containing 1.2 µg of each polyamine was derivatized with dansyl chloride [5]. A volume of the dansylated standards containing 60 ng of each standard was applied to HPTLC plates.

### DNA and protein sequence analysis

DNA and protein sequences were obtained from GenBank and sequence alignments made with Clustal Omega [12] at EMBL-EBI (https://www.ebi.ac.uk/jdispatcher/). The genomic contexts of the *hss* and *casdh* genes were obtained from Microbes Online (http://www.microbesonline.org/) using the Gene Tree function with the *S. meliloti* 1021 *hss* (*smc04016*) or *casdh* (*smb21630*) as the query sequences.

### PCR amplification and DNA manipulations

PCR primers (Table S1, available in the online Supplementary Material) were used in reactions with Dream Taq PCR master mix (Thermo Fisher) to amplify genes or analyse genetic constructs. The PCR programme routinely used included a denaturing step at 95 °C for 1 min followed by 30 cycles of 95 °C for 1 min, 56 °C for 1 min and 72 °C for a time appropriate for the length of the DNA being amplified. A final elongation step was made at 72 °C for 10 min. DNA isolation, restriction digests, cloning and transformation were performed by standard methods [11] or using commercially available kits. Bacterial conjugations were done as described previously [5].

### Mutant construction

To construct the *S. meliloti* Rm8530 *hss* and *casdh* mutants, their respective ORFs with ~600 flanking nt were amplified from *S. meliloti* 1021 genomic DNA using primer pairs hssmut1F/hssmut1R and casdh1/casdh2, respectively (Table S1).

The *hss* PCR product was cloned into pTZ57R/T to generate pTZ-hss (Table 1), using *E. coli* DH5*α* as a host for subsequent manipulations. The plasmid was digested with *Sal*I, three sites for which occur in the insert, resulting in the removal of 810 nt of the *hss* sequence. A loxP Sp cassette cut with *Sal*I was ligated into *Sal*I-digested pTZ-hss to generate plasmid pTZ-hss::loxSp. The *hss*::loxSp insert was excised from pTZ-hss::loxSp using *Xba*I and *Bam*HI and inserted into the positive-selection suicide vector pJQ200SK+, cut likewise, to form pJQ-hss::loxSp. This plasmid was conjugated from DH5*α* into *S. meliloti* Rm8530 and double recombinants isolated by sucrose selection [5] to obtain the *hss* null mutant, which was confirmed by PCR using primers hssIntF and hssIntR (Table S1) and was named 8530-hss (Table 1).

The *S. meliloti casdh* PCR product was cloned into vector pCR2.1-Topo and transformed into *E. coli* Mach1 cells following the manufacturer’s instructions. Plasmids containing the insert were confirmed by restriction enzyme digestions. The *casdh* insert was excised with *Bam*HI and *Xho*I and cloned into plasmid pJQ200SK+ cut likewise. An ΩSp fragment was inserted into the *Sma*I site of the *casdh* gene in pJQ200SK+ to generate plasmid pJQ-casdh::ΩSp. Double recombinants in *S. meliloti* Rm8530 were obtained as described for the *hss* mutagenesis. The *casdh* null mutant obtained was confirmed by PCR using primers casdh1 and casdh2 (Table S1) and was designated 8530-casdh (Table 1).

The Rm8530 *hss casdh* double mutant was constructed as described previously [5]. Briefly, the loxP Sp interposon in the *hss* gene in 8530-hss was deleted by introducing plasmid pBBRMCre, which expresses the loxP-specific Cre recombinase, into the mutant. The desired loxP Sp deletion and pBBRMCre plasmid-cured strain was selected by screening for the Sm^r^ Sp^s^ and Sm^r^ Gm^s^ phenotypes, respectively. In the second step, plasmid pJQ-casdh::ΩSp was introduced into the loxSp-deleted 8530-hss mutant to obtain double mutant 8530-hss-casdh by selection for sucrose sensitivity. The ~2.2 kb increase in the *casdh* gene length due to the insertion of ΩSp was confirmed by PCR with primers casdh2 and smb21630F (Table S1).

### Construction of plasmids for genetic complementation

Plasmid pBB5-hss (Table 1) containing the *hss* gene and flanking regions was constructed by digesting plasmid pTZ-hss with *Xba*I and *Apa*I and ligating the liberated insert into vector pBB5 (Table 1). Plasmid pBB5-casdh containing the *casdh* gene and surrounding nts was obtained by liberating the *casdh* fragment from pTopo-casdh with *Bst*XI and ligating it into plasmid pBB5.

### Construction and assay of transcriptional fusions with *β*-glucuronidase (*gusA*)

To amplify the *hss* promoter region for insertion into the *gusA* fusion vector pBB53, a 630 nt fragment containing 404 nt upstream of the Rm8530 *hss* start codon and 233 nt of the coding sequence was amplified by PCR using primers hssF and hssR (Table S1). The *casdh* gene promoter region containing 82 nt upstream of the start site and 189 nt of the coding sequence was amplified from strain Rm8530 using primers smb21630F and smb21630R (Table S1). Both promoter regions were amplified using 1 cycle of 94 °C, 2 min followed by 30 cycles of 94 °C, 30 s; 58 °C, 30 s; and 68 °C, 45 s. The final extensions were at 72 °C for 10 min. The PCR products were cloned into pCR2.1-Topo (Table 1). The *hss* and *casdh* promoter inserts were excised from pCR2.1-Topo using the flanking *Kpn*I and *Xba*I sites of the vector and cloned into pBB53 to create plasmids pBB53hss::gusA and pBB53casdh::gusA, respectively (Table 1). The correct transcriptional orientations of the inserts with respect to *gusA* were confirmed by restriction enzyme digestions and by PCR analysis utilizing primer p53lw (reverse primer specific for *gusA*; Table S1) and the relevant forward primer for the cloned *hss* or *casdh* promoters. The *gusA* fusion plasmids were transferred to *S. meliloti* 1021 and Rm8530 by triparental mating. For Gus assays, triplicate cultures for each treatment were grown in MMSA in the absence or presence of 0.1 mM Put, Spd or HSpd for 16 h at 30 °C with shaking at 200 r.p.m. *β*-Glucuronidase (Gus) activity was determined in two independent experiments with triplicate biological replicates and two technical replicates for each culture in each experiment. Gus activity was measured by the production of *p*-nitrophenol from the *p*-nitrophenyl *β*-d-glucuronide substrate, and specific activities were calculated based on total protein. One unit of activity is defined as the production of 1 nmol of product min^−1^ mg protein^−1^ ([5].

### Biofilm formation, autoaggregation and EPS assays

Biofilm, EPS and cell autoaggregation assays were done as described in [3]. Briefly, biofilm formation in the presence or absence of 0.1 mM added polyamine was determined with cultures grown in borosilicate glass tubes. EPS was estimated by measuring the carbohydrate content of supernatants prepared from the biofilm tube cultures using the anthrone assay. Cell autoaggregation was determined with 24 h static cultures in MMSA as the ratio of the OD_595_ of cells remaining in suspension to that of the total cells in the culture by the formula 100[1-OD_suspended cells_−OD_total cells_].

### Motility assays

Swimming and swarming assays were performed in Bromfield medium plates containing 0.3% or 0.6% Noble agar (Sigma), respectively. For each assay, three inoculations were made in each of three plates for each strain and condition, and experiments were done twice. Exogenous polyamines were added at 0.1 mM for swarming assays and 0.01 mM for swimming assays. Plates were inverted and incubated at 30 °C for 72 h before measuring swarming and swimming displacement zones. Swarming zones were measured by taking the average of two sides of a rectangle that framed the zone using the Macintosh Preview program rectangular selection tool. Swimming motility was measured by determining the diameters of the displacement zones [3].

### Symbiosis assays

Symbiotic parameters [6] were determined 40–44 days after inoculating alfalfa (*M. sativa*) var*.* Cuf 101 plants with Rm8530, 8530-hss, 8530-casdh or 8530-hss-casdh. For Rm8530 and the single mutant strains, results are from two independent experiments with ten plants analysed for all parameters except acetylene reduction, for which six plants were used. For 8530-hss*-*casdh, results are from three independent experiments with the number of plants analysed per experiment as stated above. The results were analysed by the LSD (Least Significant Difference) Fisher test.

## Results

### Sequence and genomic context of the *S. meliloti hss* and *casdh*

*S. meliloti* strain Rm8530 (a derivative of 1021 with a functional *expR* gene) was used as a wild-type [13,14]. Because Rm8530 is otherwise isogenic to 1021, we used the published 1021 genome sequence [15] for *in silico* analyses and genetic manipulation of Rm8530. ExpR is required for full quorum-sensing ability in *S. meliloti* [14].

Homologues of *hss* occur in many *α*-, *γ*- and *δ*-protobacteria, including nitrogen-fixing rhizobia [7]. The amino acid sequence of the *S. meliloti* 1021 Hss (SMc04016) is 73.7 % identical to that of the biochemically and structurally characterized Hss from the alphaproteobacterium *Blastochloris viridis* and conserves the eight key catalytic or substrate binding residues identified in that enzyme [16] (Fig. S1).

The *S. meliloti* 1021 *hss* gene does not lie near other genes involved in polyamine metabolism (Fig. S2). This contrasts some other species of proteobacteria in which polyamine-related genes are encoded near *hss*. For example, *odc* genes lie near *hss* in the alphaproteobacterial marine species *Stappia aggregata* (now *Labrenzia aggregata*; the *odc* is annotated as COG-LysA), the plant growth-promoting rhizobacteria *Azospirillum* (annotated as speF) and the pathogenic acetic acid bacterium *Granulibacter bethesdensis* (annotated as COG-LdcC). Agmatinase (*speB*) occurs in *Sagittula stellata* (alphaproteobacteria) and a putrescine transporter (*potE*) is present in *Shewanella amazonensis* (gammaproteobacteria) (Fig. S2). The genomic context around *hss* is conserved in *S. meliloti* 1021 and *Sinorhizobium medicae* WSM419 and largely conserved in *Rhizobium* sp. NGR234 (now *Sinorhizobium fredii* NGR234) (Fig. S2) and in strains *S. meliloti* strains GR4, RM017, Rm41, SM11, AK83 and BL225C (not shown). Genes flanking *hss* in these species include a predicted diguanylate cyclase (cyclic di-GMP synthesis; labelled COG2199 in Fig. S2), a 5’-nucleotidase (UDP-glucose catabolic enzyme; COG-UshA) and ferrochelatase (haem synthesis; *hemH*) (Fig. S2).

We previously proposed that Spd in *S. meliloti* was synthesized from Put by the reactions catalysed by Casdc and Casdh [7] (Fig. 1a). Casdh (SMb21630) is annotated as a saccharopine dehydrogenase family protein. We originally designated the *casdh* gene described here as a carboxynorspermidine dehydrogenase (Cansdh) based on the annotation of the neighbouring gene as carboxynorspermidine decarboxylase (*nspC*) [5]. Although Casdh and Cansdh are homologous enzymes, Casdh participates in Spd synthesis (Fig. 1a), while Cansdh condenses aspartate semialdehyde and 1,3-diaminopropane to form carboxynorspermidine as part of the NSpd synthesis pathway (Fig. 1b). Because the results presented here show that *smb21630* is essential for Spd biosynthesis*,* the gene and enzyme product are referred to here as *casdh* and Casdh, respectively. Deduced protein sequence alignments of the *Helicobacter pylori* and *S. meliloti* Casdh sequences show the conservation in *S. meliloti* of key amino acid residues identified in the *H. pylori* enzyme [17] (Fig. S3). We do not know whether Casdh participates in NSpd synthesis in *S. meliloti* Rm8530.

The *S. meliloti* 1021 *casdc* and *casdh* genes are predicted to be transcribed as a two-gene operon (Microbes Online). The two genes are separated by 43 nt and a potential Shine-Dalgarno sequence (AAGGAG) begins 18 nt upstream of the predicted start codon of the second gene, *casdh*. This indicates that *casdh* can be expressed independently of *casdc*, consistent with our transcriptional expression and genetic complementation data. As expected, verified Spd-producing *Sinorhizobium* and *Agrobacterium* contain the *casdh-casdc* operon, which is absent from rhizobia that do not synthesize Spd [7,10] (Fig. S4).

In *S. meliloti* 1021 and *S. medicae* WSM419, a cluster of genes encoding proteins that participate in fatty acid beta-oxidation is encoded upstream of the *casdc-casdh* operon (Fig. S4). Genes whose products function in phenylacetic acid (PAA) catabolism lie further upstream. The genomic contexts of *casdc-casdh* in the other bacteria represented in Fig. S4 are quite variable.

### Polyamine production by the *S. meliloti* wild-type and mutant strains

**Fig. 2.**
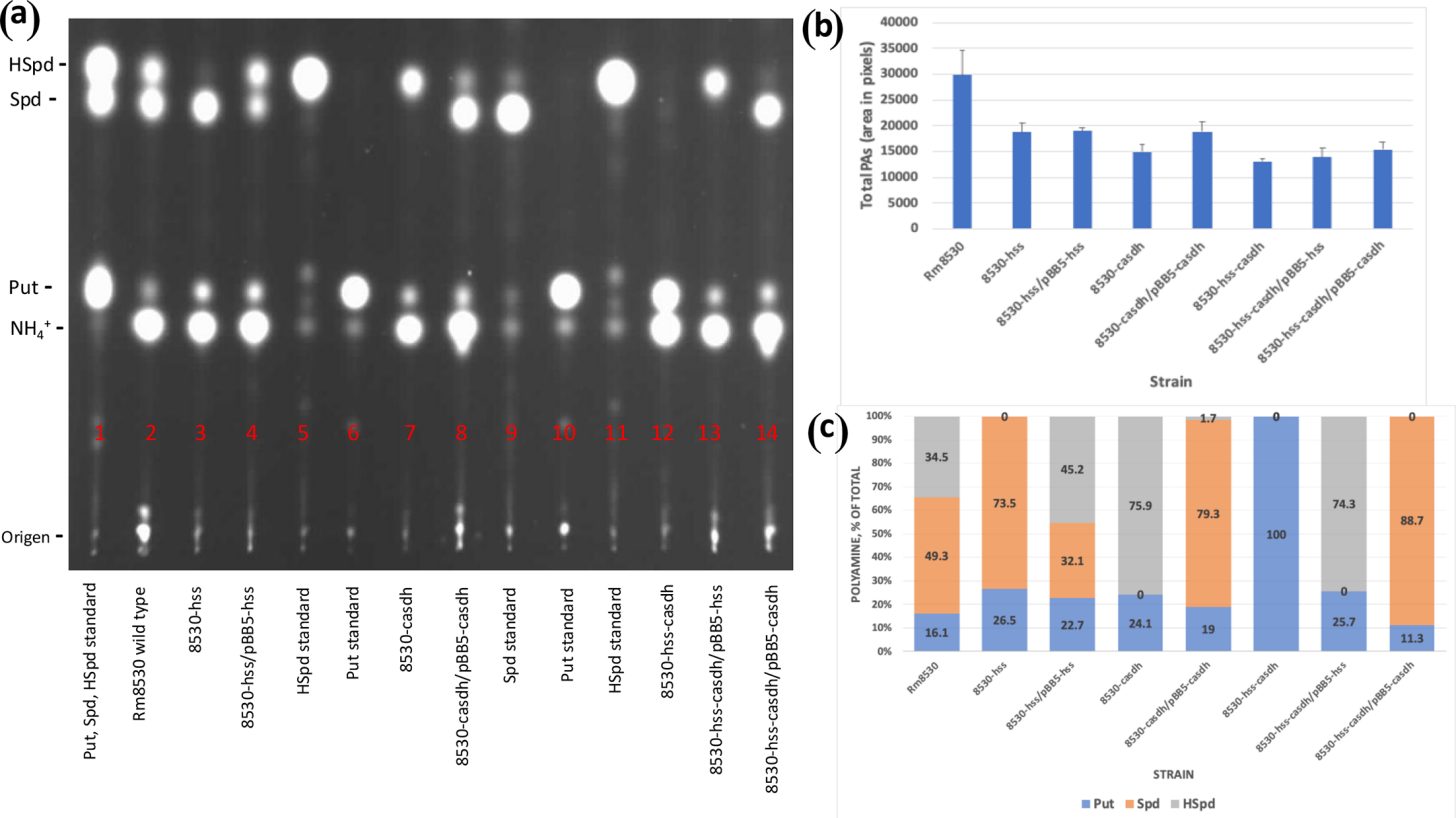
Profiles of endogenous free polyamines present *S. meliloti* strains. (**a**) Resolution of dansyl-polyamine derivatives of endogenous *S. meliloti* polyamines by HPTLC. Rm8530 wild-type and *hss* and *casdh* mutants, with or without genetic complementation, were grown in minimal medium cultures and polyamines extracted, derivatized and separated by HPTLC [5]. A representative plate from one of two independent experiments is shown. Lanes 1, 5, 6 and 9–11 contain dansyl polyamine standards: Put (P), Spd (S) and HSpd (H), whose position is indicated to the left of the image. Lane 2, Rm8530 wild-type; lane 3, *hss* mutant; lane 4, *hss* mutant complemented with *hss*; lane 7, *casdh* mutant; lane 8, *casdh* mutant complemented with *casdh*; lane 12, *hss casdh* double mutant; lane 13, double mutant complemented with *hss*; lane 14, double mutant complemented with *casdh*. Densitometry of dansyl-polyamines resolved by HPTLC was used to determine the total polyamine content (**b**) (mean±sd for two technical repetitions per sample) and content of individual polyamines (**c**) expressed as the per cent of the total polyamines in the sample.

Dansyl derivatives of endogenous polyamines from cells of the *S. meliloti* wild-type and mutant strains grown in MMSA medium were resolved by HPTLC (Fig. 2a), and spot intensities were determined by densitometry. Sample loading was normalized to culture cell densities, making it possible to compare relative polyamine levels among the different samples. The total polyamine content for a given sample (Fig. 2b) was calculated by summing the spot quantities for Put, Spd and HSpd. The relative proportions of the three polyamines are shown in Fig. 2(c). NSpd was not detected in any sample, consistent with previous results showing its confinement to the bound polyamine fraction [5].

Wild-type strain Rm8530 produced 180% the level of total polyamines as the *hss* and *casdh* mutants without or with genetic complementation (Fig. 2b). In the wild-type, Put, Spd and HSpd accounted for ~16, 49 and 35% of the total (Fig. 2c), consistent with previous results [5].

As expected, *hss* mutant 8530-hss did not produce HSpd. It made 150 and 160% the proportions of Spd and Put as the wild-type (Fig. 2a, c). In comparison to the wild-type, strain 8530-hss/pBB5-hss modestly overproduced HSpd (130%) as a proportion of its total polyamine content and produced 35% lower proportion of Spd and 140% greater proportion of Put (Fig. 2c). Despite the differences in the relative levels of the polyamines between 8530-hss without or with plasmid pBB5-hss, the total quantity of polyamines produced by each was very similar (Fig. 2b).

Our prediction that Casdh was required for Spd production was confirmed by the inability of 8530-casdh to produce this polyamine. The level of HSpd in 8530-Casdh, expressed as a proportion of the total polyamine content, was 220% of that in the wild-type strain. The overproduction of HSpd in 8530-casdh was similar to the level of Spd overproduction by 8530-hss. The introduction of pBB5-casdh into 8530-casdh (strain 8530-casdh/pBB5-casdh) caused essentially all of the HSpd produced by the uncomplemented mutant to be replaced with Spd (Fig. 2c).

Double mutant 8530-hss-casdh produced only Put, and the quantity of this sole polyamine was present at 270% that of the wild-type (Fig. 2a). The double mutant complemented with pBB5-hss produced a very high proportion of HSpd and, as expected, no Spd. The introduction of pBB5-casdh into the double mutant resulted in the production of the highest proportion of Spd in any of the strains and a reduction of the proportion of Put to the lowest level among the strains (Fig. 2c).

### Growth of *S. meliloti hss* and *casdh* mutants

**Fig. 3.**
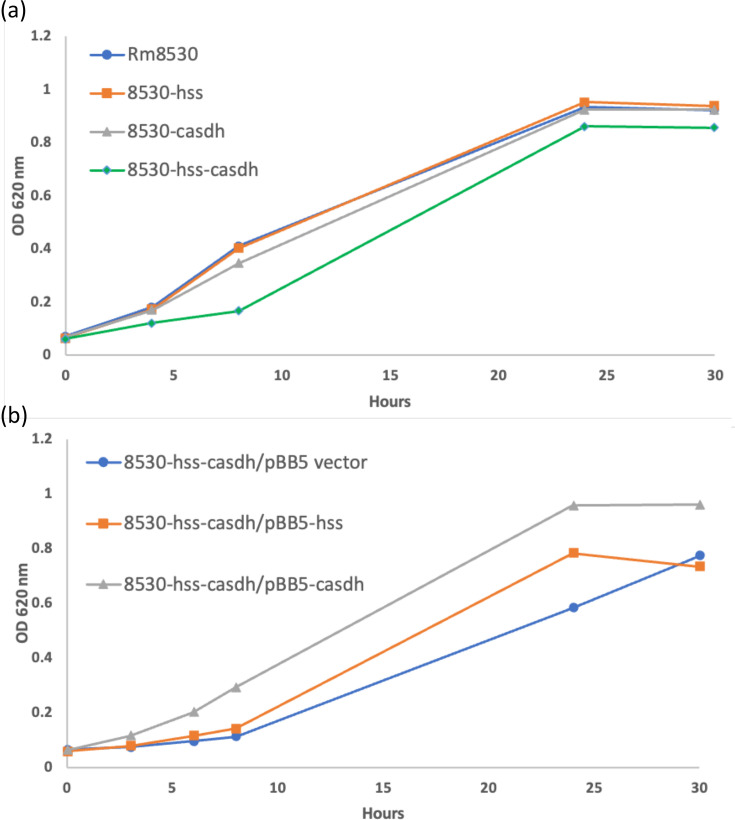
Growth of *S. meliloti* strains in the second subculture in MMSA. (**a**) Growth of the Rm8530 wild-type and the *hss* and *casdh* single and double mutants derived from it. Values are the means for two independent experiments with two biological replicates each. sd differed from the means by less than 15%. (**b**) Growth of the *hss casdh* double mutant containing the pBB5 empty vector or pBB5 with the *hss* or *casdh* gene. Values are the means for two independent experiments with two biological replicates each and sd differed less than 10% from the means.

Growth kinetics of the Rm8530 wild-type, 8530-hss, 8530-casdh and 8530-hss*-*casdh were determined in the second subculture in MMSA containing biotin. Subculturing in minimal medium was done to deplete the cells of polyamines taken up from the rich medium precultures, and biotin was added because *S. meliloti* is a biotin auxotroph [18]. The wild-type and 8530-hss had very similar growth (Fig. 3a), with the mutant’s generation time (*g*) being 3–4% lower than those of the wild-type between 4 and 8 h (*g*=13.6 and 13.2 h, respectively) and between 8 and 24 h (*g*=13.5 and 12.9 h, respectively). In contrast, 8530-casdh grew more slowly than the wild-type and 8530-hss from 4 to 8 h (*g*=15.5 h) but more rapidly during the 8–24 h interval (*g*=11.3 h), ultimately reaching a comparable cell density. The 8530-hss-casdh double mutant exhibited a very slow initial growth rate (*g*=35.3 h) and a marked delay in entering the exponential phase. However, once in exponential phase, its growth rate (*g*=6.8 h) exceeded that of the other strains, although it stopped growing at a lower final cell density (Fig. 3a). These observations highlight that the calculated generation times vary across different portions of the growth curve and indicate that *hss* and *casdh* contribute additively or synergistically to normal growth under these conditions. To further establish the relative contributions of Hspd and Spd to growth, we genetically complemented 8530-hss-casdh with the *hss* or *casdh* gene expressed from plasmid pBB5, or with pBB5 lacking an insert as a control (Fig. 3b). The empty vector control had a long lag phase, relatively slow growth rate and low final cell density, while the *hss*-complemented mutant grew similarly to the wild. The *hss*-complemented mutant’s growth was intermediate between that of the empty vector control and the *casdh* complemented strain (Fig. 3b).

### Effect of exogenous polyamines and quorum sensing on *hss* and *casdh* expression

Many phenotypes affected by polyamine metabolism, including motility, EPS production and biofilm formation, are also modulated by quorum sensing (QS) in *S. meliloti* [3]. *S. meliloti* strains Rm8530 and 1021 are identical except that strain 1021 lacks a functional copy of the transcriptional regulator gene *expR*, which is required for full QS activity [13,14]. Using *gusA* transcriptional fusions to monitor *hss* and *casdh* expression in these strains, we found that the transcription of *hss* in strain Rm8530 was less than half that in strain 1021 (453±28 versus 954±2.5). The expression of *casdh* in strain Rm8530 was about one-third lower than that in strain 1021 (59±7 versus 37±0.5). Despite the much higher expression of *hss* and *casdh* in strain 1021, the levels of intracellular polyamines do not markedly differ between this strain and Rm8530 [5]. In Rm8530, the cellular content of HSpd is somewhat lower than that of Spd despite the higher transcriptional expression of the *hss* gene (Fig. 2c).

**Fig. 4.**
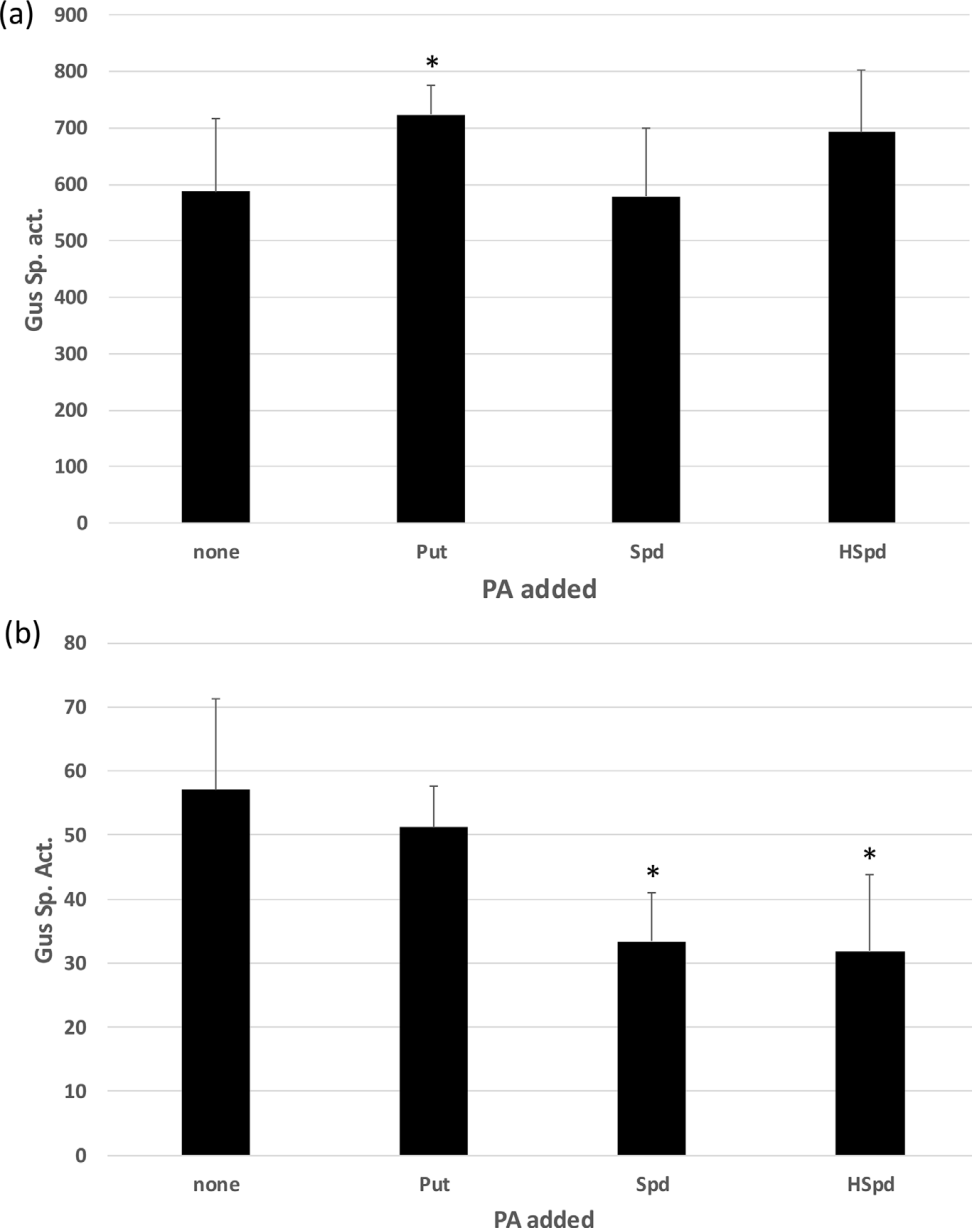
Expression of hss (a) and casdh (b) in Rm8530 determined with transcriptional fusions to gusA. Strains were grown in MMSA without or with 0.1 mM exogenous Put, Spd or HSpd. Values are the mean±sd from two independent experiments with three biological replicates each. Values differing from the unsupplemented controls at p ≤ 0.05 are marked with an asterisk.

We examined the expression of the *hss* and *casdh* genes in Rm8530 in the presence of exogenous Put, Spd or HSpd (Fig. 4a, b). A statistically significant change in *hss* expression occurred in the presence of Put, its substrate. Unexpectedly, *hss* transcription had a similar, although not statistically significant, increase in the presence of its product, HSpd (Fig. 4a). The transcription of *casdh* was lowered to ~56% the control level by exogenous Spd or HSpd (Fig. 4b). It should be noted that Put, when added exogenously to cultures, alters the levels of Spd and HSpd, both of which are derived from it. In independent experiments, we quantitated the effect of supplementation with 0.1 mM Put on the levels of Spd and HSpd using HPTLC and densitometry. Results from two independent experiments with a total of three biological replicates showed that in cultures grown with 0.1 mM Put, the levels of Put, Spd and HSpd were 46±13, 83±7 and 139±29% those of the cultures grown without Put. The total of Put+Spd+HSpd produced by the cultures did not differ greatly (pixel areas: control, 31,770±2,477; 0.1 mM exogenous Put, 28,156±3,787). Similarly, we previously reported that Rm8530 grown in MMSA with 1 mM Put had decreased Put and Spd levels and increased HSpd content [5].

### Swimming and swarming motility are reduced in 8530-hss and 8530-hss-casdh

**Fig. 5.**
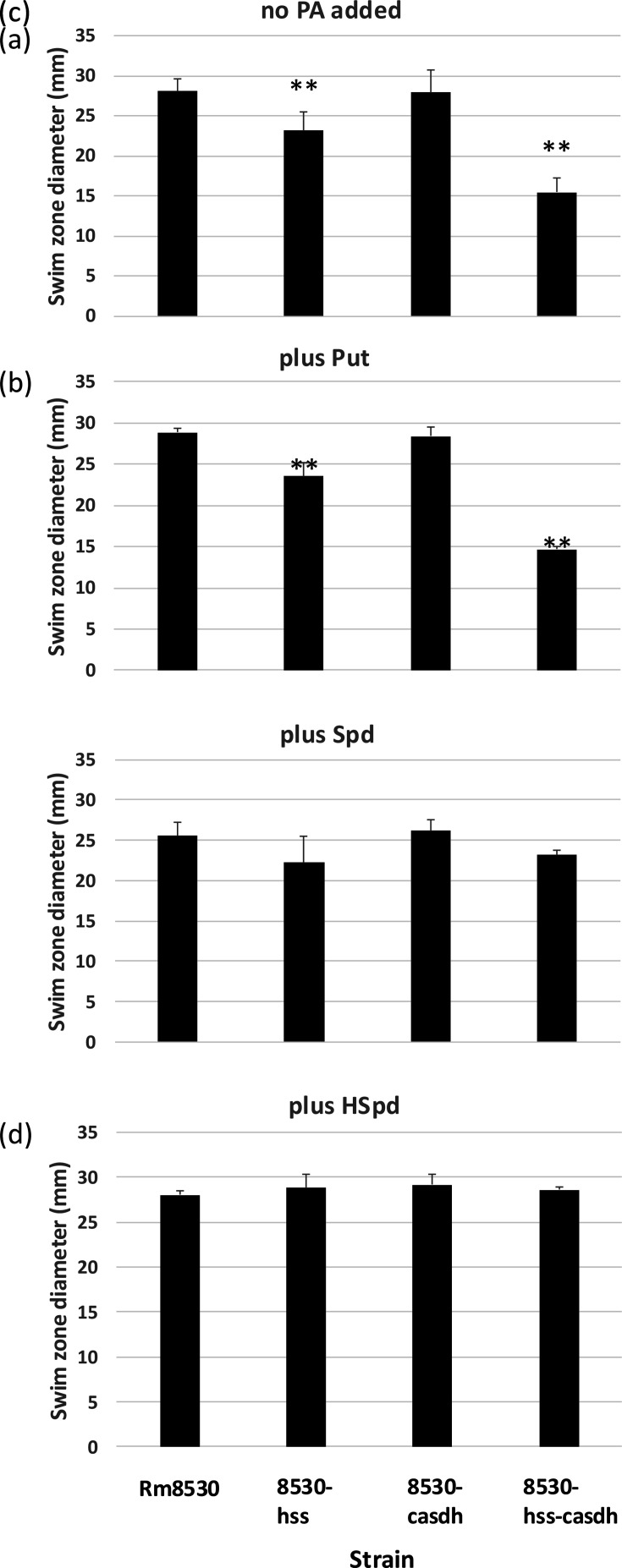
Swimming motility of *S. meliloti* Rm8530 wild-type, *hss* and *casdh* single mutants and *hss casdh* double mutant without added polyamine (**a**) or with chemical complementation using 0.01 mM final concentrations of exogenous Put (**b**), Spd (**c**) or HSpd (**d**). Values are the mean±sd from two independent experiments with nine biological replicates each (*n*=18). Values statistically different from the wild-type control are shown by *P≤0.05 and **P≤0.001.

Swimming motility is the movement of individual bacteria through a liquid or semi-solid environment. Our assays for the swimming motility of *S. meliloti* in semi-solid agar were determined under control conditions (no added polyamine) or with 0.01 mM added Put, Spd or HSpd (Fig. 5). Under control conditions, swimming of 8530*-*hss and 8530*-*hss-casdh was reduced 21 and 38%, respectively, relative to the wild-type. Swimming motility in 8530-casdh was identical to that of the wild-type (Fig. 5a). Put supplementation (Fig. 5b) failed to complement the phenotypes, as all strains behaved similarly to those under control conditions. In contrast, assays with either Spd or HSpd added (Fig. 5c) resulted in the restoration of motility to 8530-hss and 8530-hss-casdh (Fig. 5d).

**Fig. 6.**
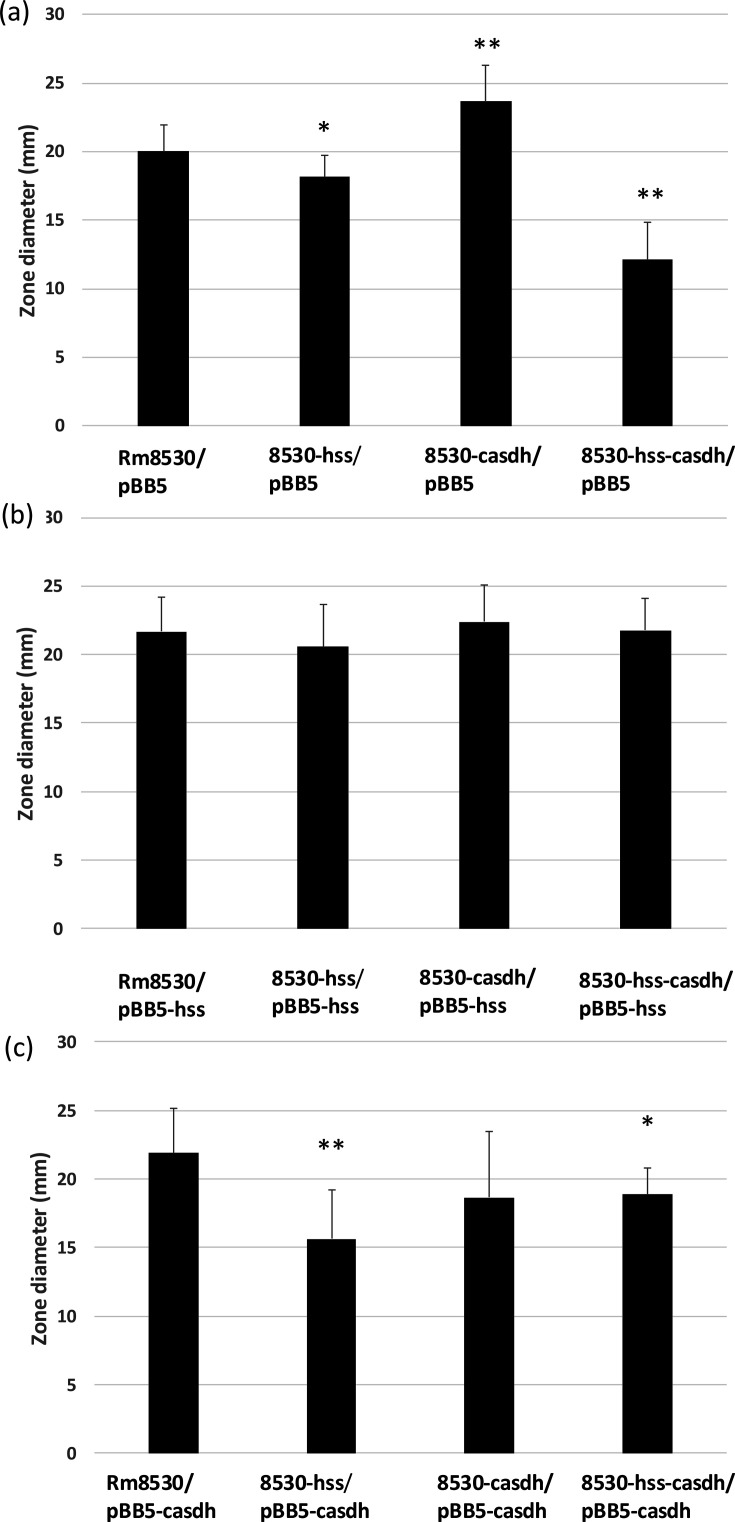
Swimming motility of *S. meliloti* Rm8530 wild-type, *hss* and *casdh* single mutants and *hss casdh* double mutant genetically complemented with pBB5 empty vector (**a**) or pBB5 containing the cloned *hss* (**b**) or *casdh* (**c**) gene. Values are the mean±sd from two independent experiments each with nine biological replicates (*n*=18). Values statistically different from the wild-type control are shown by *P≤0.05 and **P≤0.001.

For genetic complementation, we introduced vector pBB5 without insert or with the cloned *hss* or *casdh* genes into the mutants, which resulted in the production of the polyamine product of the introduced gene (Fig. 2a). Swimming assays were done without added polyamines (Fig. 6). In assays with the strains containing the pBB5 empty vector, 8530-hss/pBB5 had a slight but statistically significant reduction (9%) in swimming motility versus control strain Rm8530/pBB5, while the 8530-casdh/pBB5 showed an 18% increase in comparison to the control. In comparison to Rm8530/pBB5, 8530-hss-casdh/pBB5 had 40% lower swimming motility (Fig. 6a).

When the strains were complemented with pBB5-hss, both the *hss* and *casdh* single mutants and the double mutant swam like the wild-type. Thus, *hss* expressed in trans fully complemented the swimming defects of the 8530-hss and 8530-hss-casdh mutants (Fig. 6b). For the strains complemented with *casdh*, 8530-hss/pBB5-casdh had 28% less motility relative to Rm8530/pBB5-hss (Fig. 6c), a motility decrease greater than that seen in the 8530-hss/pBB5 empty vector control (Fig. 6a). This indicates that Spd overexpression reduced the swimming ability of 8530-hss. In comparison to the 8530-casdh/pBB5 vector control or 8530-casdh/pBB5-hss (Fig. 6a, b), Spd overexpression in 8530-casdh/pBB5-casdh caused lower swimming motility (Fig. 6c). When expressed in 8530-hss-casdh, the *casdh* gene partially complemented swimming motility, increasing it to 86% that of the wild-type complemented pBB5-casdh (Fig. 6c).

**Fig. 7.**
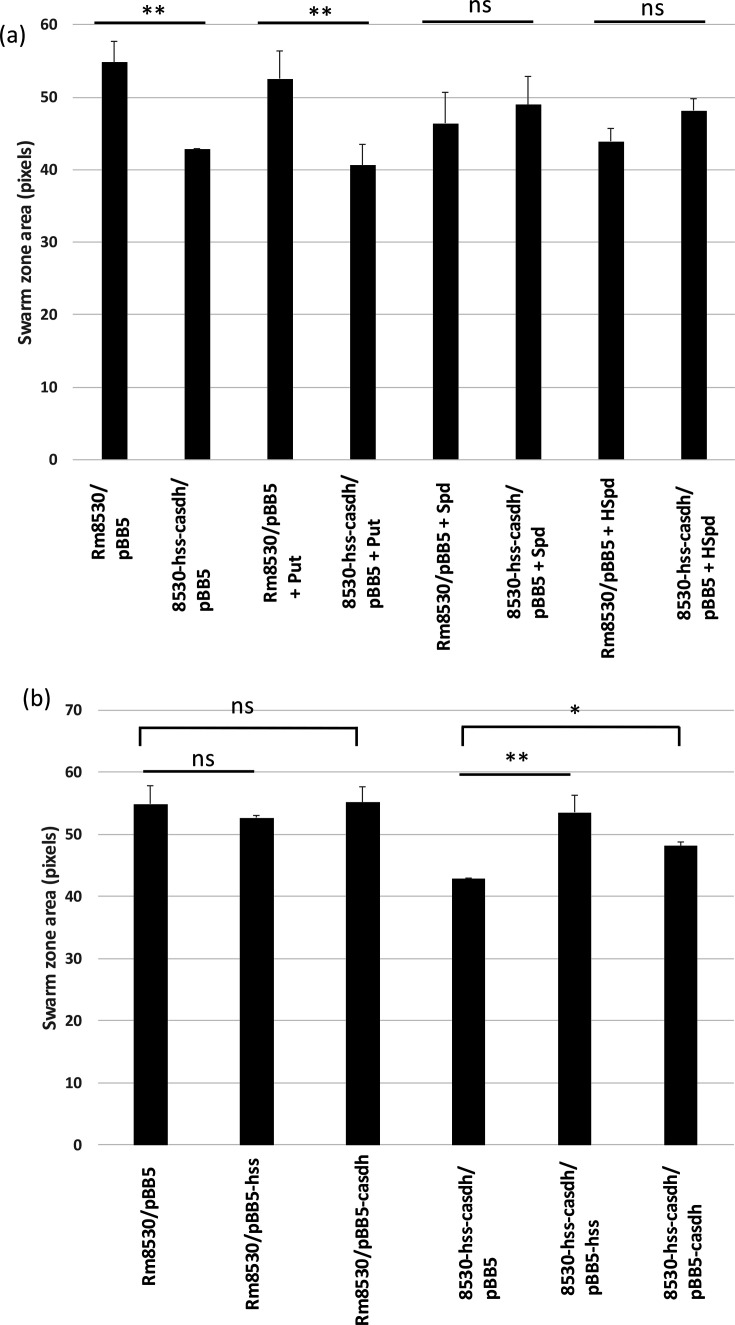
Swarming motility of the Rm8530 wild-type and *hss casdh* double mutant. (**a**) Assays performed without or with chemical complementation using 0.1 mM final concentrations of exogenous Put, Spd or HSpd. (**b**) Genetic complementation with pBB5 empty vector or pBB5 containing the *hss* or *casdh* gene. Values are the mean±sd from two independent experiments each with nine biological replicates (*n*=18). Statistically, differences between the double mutant and wild-type are shown by *P≤0.05 and **P≤0.001. ns, no significant difference.

Swarming motility is a coordinated, multicellular movement of bacteria over a solid or semi-solid surface, such as medium containing 0.6% agar. The effects of endogenous synthesis of Spd or HSpd on swarming motility were compared in Rm8530/pBB5 and 8530-hss-casdh/pBB5 with or without chemical complementation with 0.1 mM Put, Spd or HSpd (Fig. 7a). 8530-hss-casdh/pBB5 had over 20% less swarming motility than Rm8530/pBB5 without added polyamine and in assays containing Put (Fig. 7a). In contrast, the reduced swarming in the double mutant was prevented by supplementation with Spd or HSpd (Fig. 7a). Assays of the wild-type and double mutant complemented with the cloned *hss* or *casdh* gene in the absence of exogenous polyamines are shown in Fig. 7(b). Swarming motility was not significantly different in Rm8530 containing the empty vector or with the vector expressing either the *hss* or *casdh* gene. In contrast, 8530-hss-casdh/pBB5 swarmed 22% less than the Rm8530/pBB5. Swarming was completely restored in 8530-hss-casdh/pBB5-hss, while complementation of the double mutant with the cloned *casdh* gene increased its swarming to 88% that of the wild-type containing the empty vector (Fig. 7b).

### Biofilm formation, EPS production and autoaggregation were not significantly affected in the *hss* and *casdh* mutants

**Fig. 8.**
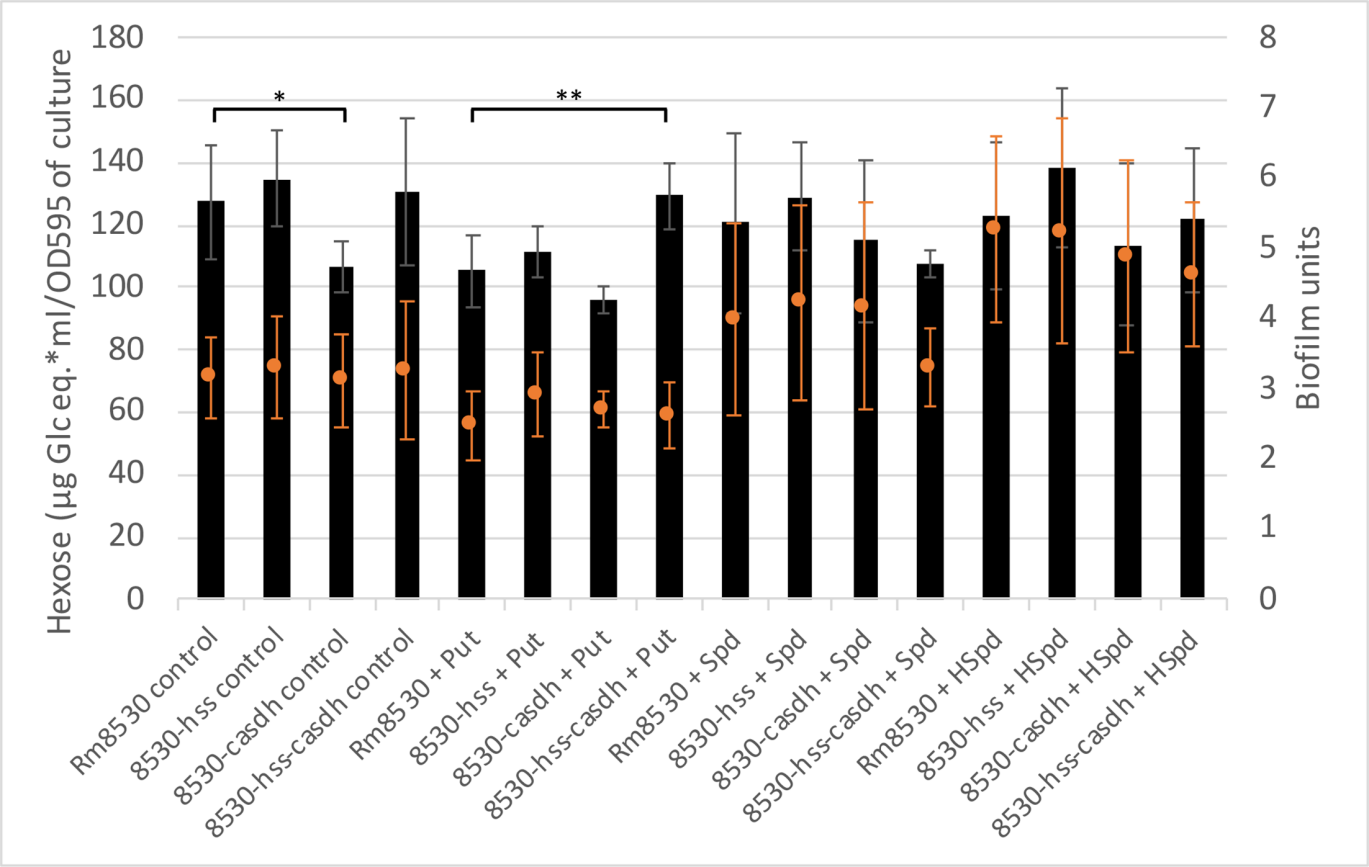
The production of EPS (black bars) and biofilm (red dots within bars) by the Rm8530 wild-type and mutants grown in MMSA without (control) or with 0.1 mM Put, Spd or HSpd. Biofilm formation of 3-day tube cultures was calculated as the ratio of the OD_595_ of planktonic cells to the A_595_ of CV-stained biofilms. Biofilm values are the mean±sd for three independent experiments in which each strain under each condition was assayed in triplicate (*n*=9). No significant differences in biofilm formation occurred between the wild-type and mutants receiving the same treatment. EPS content of supernatants (units of µg glucose equivalents mL⁻¹ OD₅₉₅⁻¹) was determined with the anthrone assay. EPS data are the mean±sd for two independent experiments in which each strain under each condition was assayed in triplicate. Data were analysed by one-way ANOVA with Tukey post-test. Significant differences in EPS production between a mutant and the wild-type strain receiving the same treatment are indicated above the brackets: *P≤0.05 and **P≤0.01.

Biofilm formation by Rm8530 and the *hss* and *casdh* mutants was determined in MMSA cultures without or with 0.1 mM exogenous polyamine (Fig. 8). There were no statistically significant differences in biofilm formation between the strains within each treatment group.

To estimate EPS production, the hexose content of the supernatants from the biofilm assay cultures was measured by anthrone assay and normalized to the OD_595_ of the planktonic cells from these cultures (Fig. 8). Differences in EPS production between the mutants and wild-type were statistically significant (*P*≤0.05) in only two cases: 8530-casdh grown in the absence of polyamines produced 14% less EPS than the wild-type, and 8530-hss-casdh grown with exogenous Put made 1.23-fold more EPS than the wild-type. Biofilm formation and EPS levels did not show a consistent correlation.

In autoaggregation assays with the wild-type and *hss cansdh* double mutant, aggregation values (see Methods) for Rm8530 and 8530-hss-casdh cells were 1.55±0.21 and 1.78±0.53, respectively, for three independent experiments each with four biological replicates per strain (*n*=12). These values are not significantly different at *P*≤0.05.

### Symbiotic phenotypes of the *hss* and *casdh* single and double mutants

**Fig. 9.**
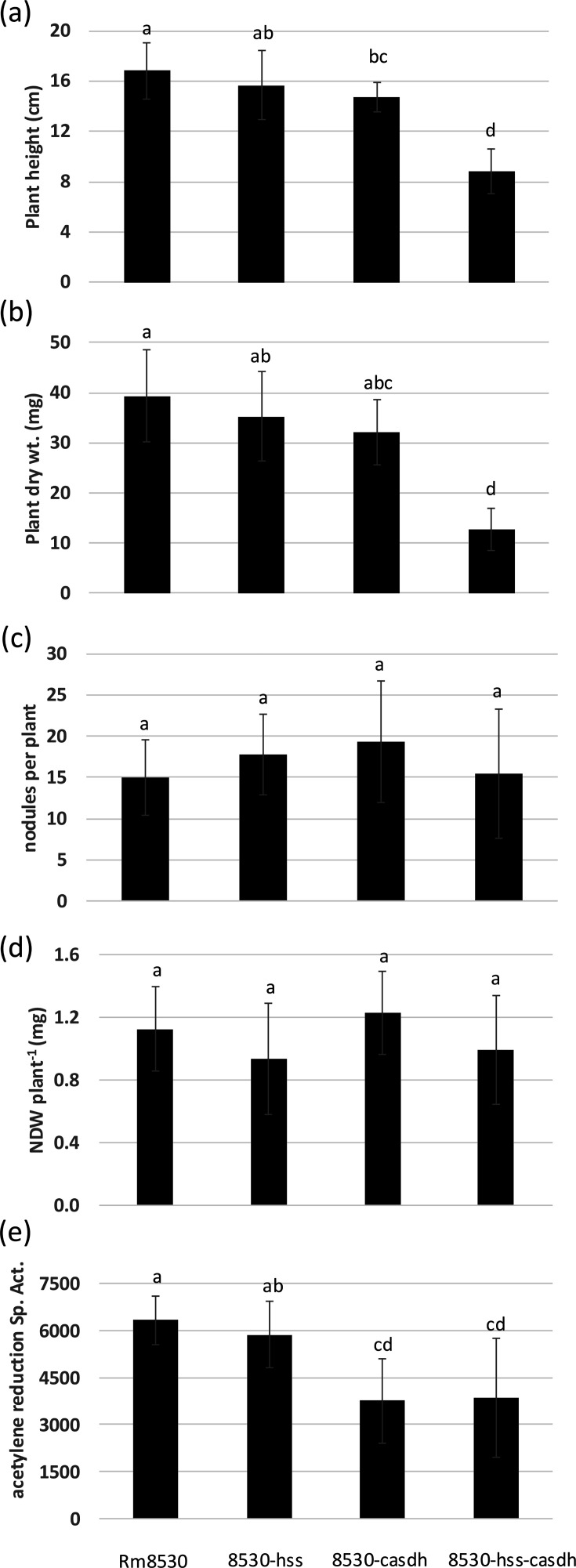
Symbiotic performance of the Rm8530 wild-type and *hss*, *casdh* and *hss casdh* mutants on alfalfa. (**a**) Plant height. (**b**) Plant dry weight. (**c**) Number of nodules per plant. (**d**) Nodule dry weight. (**e**) Acetylene reduction activity. Values are the mean±sd for two to three independent experiments as described in Methods with plant analyses performed at 40–44 days post-inoculation. Bars marked with different letters are statistically different from each other (*P*<0.05) according to the LSD Fisher test. Bars sharing at least one letter are not significantly different. For panels (**c**) and (**d**), all bars are labelled ‘a’ to indicate no significant differences among them.

The symbiotic characteristics of alfalfa *cv*. Cuf 101 plants inoculated with the Rm8530 wild-type, 8530-hss, 8530-casdh and 8530-hss-casdh mutants were determined in greenhouse experiments. Statistically significant differences in symbiotic parameters between the mutants and the wild-type were as follows. The heights of plants inoculated with the 8530-hss-casdh and 8530-casdh were 47 and 12.6% lower relative to the wild-type, while the heights of plants inoculated with 8530-hss did not differ from wild-type (Fig. 9a). The dry weight of plants inoculated with 8530-hss-casdh was one-third that of those inoculated with the wild-type, while the dry weight of plants inoculated with either of the single mutants was similar to those inoculated with the wild-type (Fig. 9b). There were no significant differences in the number of nodules formed per plant (Fig. 9c) or their dry weight per plant (Fig. 9d). Nitrogen fixation measured as acetylene reduction was ~40% lower in the 8530-casdh and 8530-hss-casdh in comparison to plants inoculated with the wild-type. Acetylene reduction by 8530-hss was similar to that of the wild-type (Fig. 9e).

## Discussion

The ability to synthesize Put and HSpd as free cytoplasmic polyamines is a common feature of symbiotic nitrogen-fixing rhizobia. *S. meliloti* is exceptional in that it also produces Spd as a free polyamine [5,7]. Given the chemical similarity between Spd and HSpd (the latter contains an additional methylene group; Fig. 1a), the main goal of the present work was to determine whether these polyamines performed either distinct or overlapping functions in determining specific phenotypes. Our functional analysis of the *S. meliloti hss* and *casdh* genes confirmed their respective roles in HSpd and Spd biosynthesis. Phenotypic analysis of the *hss* and *casdh* single and double mutants revealed both distinct and common functions of Spd and HSpd.

Both *hss* and *casdh* in *S. meliloti* are encoded near genes having potential or confirmed importance in symbiosis. The genomic context of *hss* in *Sinorhizobium* strains is largely conserved and includes a neighbouring diguanylate cyclase (DGC; *smc04015*, designated COG2199 in Fig. S2) whose inactivation in *S. meliloti* 1021 caused slower growth, increased EPS production, reduced swarming motility and lower competitiveness for alfalfa nodulation [19]. The insertional inactivation of *hss* in *S. meliloti* should not affect the expression of the neighbouring DCG gene since they are transcribed in opposite directions (Fig. S2).

The *casdc-casdh* operon is encoded near genes for fatty acid beta-oxidation and PAA catabolism (Fig. S4). The ability to catabolize PAA and perform *β*-oxidation could permit *S. meliloti* to use a broad range of organic compounds as carbon and energy sources. PAA catabolism funnels aromatic compounds into central metabolism, while *β*-oxidation degrades fatty acids to generate acetyl-CoA. Both pathways contribute to ATP production and reducing equivalents, which could support growth and survival in the soil and rhizosphere, and possibly during symbiosis [20,22].

Assays of *gusA* transcriptional fusions to *hss* and *casdh* in the QS-proficient Rm8530 and the QS-deficient 1021 genetic backgrounds showed that the expression of both genes was significantly higher in the QS-deficient strain, suggesting that a functional QS system directly or indirectly lowers the expression of these genes under the conditions tested. While polyamine signalling and QS represent distinct regulatory systems, their influence converges on several key behaviours relevant to both free-living and symbiotic lifestyles. The effect of the QS system on *hss* and *casdh* expression provides another example of the overlapping control of QS and polyamines on specific physiological processes, as we recently reported for the *S. meliloti* NspS/MbaA polyamine signalling system [3]. Whether NspS/MbaA is involved in *hss* or *casdh* regulation remains to be determined. In both the 1021 and Rm8530 backgrounds, *hss* transcriptional activity was several fold higher than that of *casdh* transcription. Despite this difference, the wild-type produces similar levels of both polyamines, indicating that biochemical or transport mechanisms control the levels of these polyamines (Fig. 2a, c).

A moderate increase in *hss* promoter activity was observed in the presence of exogenous Put (the Hss substrate) and HSpd (the Hss product) (Fig. 4a). This suggests that *hss* expression is directly or indirectly sensitive to intracellular or extracellular polyamine levels. In *Rhizobium phaseoli*, both *hss* and the gene encoding arginase, which produces the Put precursor ornithine, are transcriptionally upregulated in the presence of root exudates from corn (non-host) or bean (host) [23]. Plant root exudates contain a vast number of chemical compounds, including polyamines [1]. The expression of *casdh* was significantly and equally decreased in the presence of Spd or HSpd (Fig. 4b), and we speculate that this apparent feedback response to both polyamines might reflect the frequent overlap in their functions.

The *hss* and *casdh* single and double mutants showed a number of altered phenotypes due to their inability to synthesize one or both of these polyamines. The ability to revert these altered phenotypes by chemical or genetic complementation revealed processes that were more influenced by HSpd or Spd and others in which either polyamine would serve. Because *hss* is encoded on the chromosome and is produced by all rhizobia, we expected that it would have a more central role in cellular physiology. In contrast, *casdh* is located on symbiotic plasmid b along with many genes related to niche-specific functions including symbiosis or environmental adaptation. However, our results do not neatly conform to this prediction, as described below.

Growth is a fundamental physiological process, and we found that the absence of either Spd or HSpd caused little or no change in growth rate or yield as long as the other polyamine was present (Fig. 3a). In contrast to the wild-type and single mutant strains, the *hss casdh* double mutant had an initial phase of very slow growth and a much higher growth rate than the other strains between 8 and 24 h. We interpret this as a two-phase physiological response in which the inability to synthesize Spd and HSpd impairs early adaptation to fresh medium by affecting functions such as ribosome assembly [24], DNA topology [25] and envelope physiology [26] and thus causing an extended lag phase. The faster exponential growth that follows this lag may result from *S. meliloti* not being reliant on these polyamines for growth and, in the mutant, not having to invest resources in their synthesis. Genetic complementation of the double mutant with *casdh* decreased the lag phase and restored growth to a cell density similar to that of the wild-type (Fig. 3b). The double mutant complemented with *hss* had a long lag phase similar to the vector control and reached a final cell density between that of the *casdh*-complemented mutant and the vector control. This suggests that Spd produced by plasmid-borne *casdh* is important for both early adaptation to growth in minimal medium and for reaching a wild-type cell density. The partial complementation of the double mutant by *hss* suggests that HSpd fully restores growth of the double mutant in log phase, but not during the lag or stationary phases. Based on what is known in other bacteria, the slower growth and lower yield of 8530-hss-casdh could involve the requirement for HSpd and/or Spd in DNA replication, RNA translation, maintaining membrane stability and abiotic stress resistance [1,27, 28]. In cultures, *Rhizobium leguminosarum* bv. viciae grows poorly in the presence of the ornithine decarboxylase inhibitor *α*-DFMO, but this is partially reversed when the cultures contain exogenous NSpd, Spd or HSpd. This indicates that any of these triamines function in growth-related processes in this organism [29], consistent with our finding that restoration of HSpd or Spd synthesis by genetic complementation significantly restored the growth of the *S. meliloti hss cansdh* double mutant (Fig. 3b).

Mutants 8530-hss and 8530-hss-casdh were deficient in swimming ability, with the double mutant being the more severely affected (Fig. 5a). The 8530-casdh single mutant had wild-type swimming ability. The swimming defect in 8530-hss and 8530-hss-casdh was complemented to wild-type by either Spd or HSpd to the medium (Fig. 5c, d). In contrast, genetic complementation of the defective 8530-hss and 8530-hss-casdh swimming phenotypes was accomplished with *hss*, but not with the *casdh* (Fig. 6). These results indicate a greater importance of HSpd in full swimming motility, while Spd deficiency causes an additional decrease in swimming only in the double mutant strain that also lacks HSpd.

Swarming motility and the effect of chemical and genetic complementation were tested in the Rm8530 wild-type and 8530-hss-casdh. The double mutant showed significantly less swarming ability than the wild-type, and this deficiency was corrected equally well by exogenous Spd or HSpd or introduction of *hss* or *casdh* on a plasmid (Fig. 7). The lack of an alteration in swarming phenotype in 8530-hss contrasts the swarming defect caused by the inactivation of *hss* in *R. etli* [9], possibly because Spd synthesized by *S. meliloti* provides a compensatory function.

The *S. meliloti odc2* mutant makes very low levels of Put, Spd and HSpd and produces significantly less EPS than wild-type [6]. Surprisingly, the *hss* single mutant and *hss casdh* double mutant produced EPS similarly to the wild-type, while the *casdh* single mutant showed a modest reduction in EPS production (Fig. 8). Possibly, EPS production is modulated in response to Put, Spd and HSpd balances (or absences) in the mutants. Biofilm formation was not significantly affected in any of the mutants (Fig. 8), consistent with its being more responsive to externally supplied polyamines than endogenously synthesized ones [3,6].

The results of our symbiosis assays suggest that neither Spd nor HSpd alone or in combination is required for nodule formation or attaining nodule biomass. TnSeq analysis of the *R. leguminosarum* bv. viciae-pea interaction implicated *hss* in the microsymbiont as contributing to rhizosphere fitness and root colonization [30]. The lack of symbiotic phenotype of the *S. meliloti hss* mutant found here may be due to the ability of Spd to compensate for its absence.

Our results show that Spd biosynthesis is critical for full bacteroid function. In comparison to the wild-type, the 8530-hss-casdh double mutant had lower acetylene reduction activity in alfalfa (Fig. 9e) and also decreased plant height and dry weight (Fig. 9a, b). Plants inoculated with 8530-casdh single mutant had a lowered acetylene reduction similar to the double mutant and a very modest reduction in plant height and dry weight (Fig. 9). This indicates that a lack of endogenous Spd biosynthesis specifically impairs nitrogen fixation efficiency rather than nodule formation. The lack of endogenous Spd could affect bacteroid membrane stability, transport processes or stress resistance, thus affecting their ability to fix nitrogen even though nodulation proceeds normally.

The symbiotic phenotypes of 8530-hss-casdh are similar to those of the Rm8530 *odc2* mutant [6] in that both condition lower plant heights and dry weights and, especially, lower acetylene reduction activity (Fig. 9a, b, e). They differ in that plants inoculated with the *odc2* mutant had an increased number of nodules per plant and decreased nodule dry weights [6], parameters that were not affected in plants inoculated with the *hss* and *casdh* single mutants or the *hss casdh* double mutant.

In summary, this study shows that despite their chemical similarity, HSpd and Spd fulfil both unique and compensatory roles in *S. meliloti* physiology. Of the two, Spd appears to play the more fundamental role as it is required for normal growth and full symbiotic efficiency, while the ability to synthesize HSpd has a greater impact on swimming motility. We are currently investigating the role of *S. melilot*i endogenous polyamines, including NSpd, in modulating abiotic stress resistance.

## Supplementary material

10.1099/mic.0.001668 Uncited Supplementary Material 1.

## Abbreviations

EPS: exopolysaccharide
HPTLC: high-performance TLC
MMSA: minimal medium succinate ammonium
PAA: phenylacetic acid
PY: peptone-yeast extract
QS: quorum sensing
