# Supplementary material for: Divergent and overlapping roles of homospermidine and spermidine in Sinorhizobium meliloti physiology and symbiotic performance
Source: Microbiology (Reading). 2026 Feb 6;172(2):001668. doi: 10.1099/mic.0.001668 (PMC13293326; doi:10.1099/mic.0.001668)
Supplement: Uncited Supplementary Material 1. [file mic-172-01668-s001.pdf]

Supplementary material

Table S1. Primers used in this study.

Primer name	5'-3' nucleotide sequence	Description
casdh1	ACTTCACCGGGGACGACTAT	Forward primer for *casdh* cloning for mutagenesis
casdh2	CGATGCAGATACGCAATCAC	Reverse primer for *casdh* cloning for mutagenesis
smb21630F	GTCCGCGAATTCACCTACG	Forward primer for *casdh* promoter amplification for *gusA* fusion construction
smb21630R	CTCCAGCTTCACCTCGGTCT	Reverse primer for *casdh* promoter amplification for *gusA* fusion construction
hssmut1F	GAAAGGCTTCATTGGCGTAG	Forward primer for *hss* cloning for mutagenesis
hssmut1R	GTTGACCTTGGACTGGGTGT	Reverse primer for *hss* cloning for mutagenesis
hssIntL	CTACCCGGTTTACGGTGAGA	Forward primer for confirming presence of *hss*::loxSp
hssIntR	AGTAGGCATTCTTGGCATGG	Reverse primer for confirming presence of *hss*::loxSp
hssF	ATCAGGGCCTTGAGGTGAT	Forward primer for *hss* promoter amplification for *gusA* fusion construction
hssR	TGTAGTTGTCCCTGGTGACG	Reverse primer for *hss* promoter amplification for *gusA* fusion construction
p53lw	ACAGGACGTAACATAAGGGAC T	Reverse primer for the *gusA* gene [40]

```
B. veridis          --MTDWPVYHRIDGPIVMIGFGSIGRGTLPLIERHFAFDRSKLVVIDPSDEARK--LAEA   56
S. meliloti         MADTTYPVYGEITGPIVMIGFGSIGHGTLPLIERHFKYDKNRLIVVEPREDAKDTEIFVR   60
                       * :*** .* ************:********** :*:.:*:*::* ::*:.  :

B. veridis          RGVRFIQQAVTRDNYRELLVPLLTAGPGQGFCVNLSVDTSSLDIMELARENGALYIDTVV   116
S. meliloti         HGVRHVRAAVTRDNYKELLKPLLTEGGGQGFCVNLSVDTSSLDIIKLCRKLDVLYVDTVI   120
                    :***.:: *******:*** **** * *****************::*.*: ..**:***:

B. veridis          EPWLGFYFDPDLKPEARSNYALRETVLAARRNKPGGTTAVSCCGANPGMVSWFVKQALVN   176
S. meliloti         EPWLGFYFDAEMDNAARTNYALRETVRREKEKNPGGTTAVSTCGANPGMVSWFVKKALLN   180
                    ********* ::.  **:********   :.::******** *************:**:*

B. veridis          LAADLGVTGEEPT--TREEWARLAMDLGVKGIHIAERDTQRASFPKPFDVFVNTWSVEGF   234
S. meliloti         LADDLGLKYEEPHQDDREGWAKLMKKAGVKGVHIAERDTQRAKHPKPLNVFWNTWSVEGF   240
                    ** ***:. ***    ** **:*  . ****:**********..***::** ********

B. veridis          VSEGLQPAELGWGTFERWMPDNARGHDSGCGAGIYLLQPGANTRVRSWTPTAMAQYGFLV   294
S. meliloti         ISEGLQPAELGWGTHENWMPKNAKKHKKGCKAAIYLEQPGANTRVRSWCPTPGPQYGFLV   300
                    :*************.*.***.**: *..** *.*** *********** **   ******

B. veridis          THNESISIADFLTVRDAAGQAVYRPTCHYAYHPCNDAVLSLHEMFGSG-KRQSDWRILDE   353
S. meliloti         THNESISIADFFTVRDKDGEVSYRPTCHYAYHPANDAVLSLHEMFGNGGKAQPELHVLDE   360
                    ***********:****  *:. ***********.************.* * * : ::***

B. veridis          TEIVDGIDELGVLLYGHGKNAYWYGSQLSIEETRRIAPDQNATGLQVSSAVLAGMVWALE   413
S. meliloti         HELVDGVDELGVLLYGHAKNAYWYGSRLSLEETRRIAPYQNATGLQVTSAVLAGMVWALE   420
                    *:***:**********.********:**:******** ********:************

B. veridis          NPNAGIVEADDLDFRRCLEVQTPYLGPVVGVYTDWTPLAGRPGLFPEDIDTSDPWQFRNV   473
S. meliloti         NPKAGIVEADEMDYKRCLEVQMPYLGPVEGHYTDWTPLDGRPGLFPEDIDTKDPWQFRNI   480
                    **:*******::*::****** ****** * ******* ************.*******:

B. veridis          LVRD      477
S. meliloti         LVR-      483
                    ***
```

Fig. S1. Clustal omega alignment of amino acid residues comprising the *B. veridis* and *S. meliloti* Hss proteins. Residues essential for substrate binding or catalysis in the *B. veridis* sequence [16] are highlighted in red.

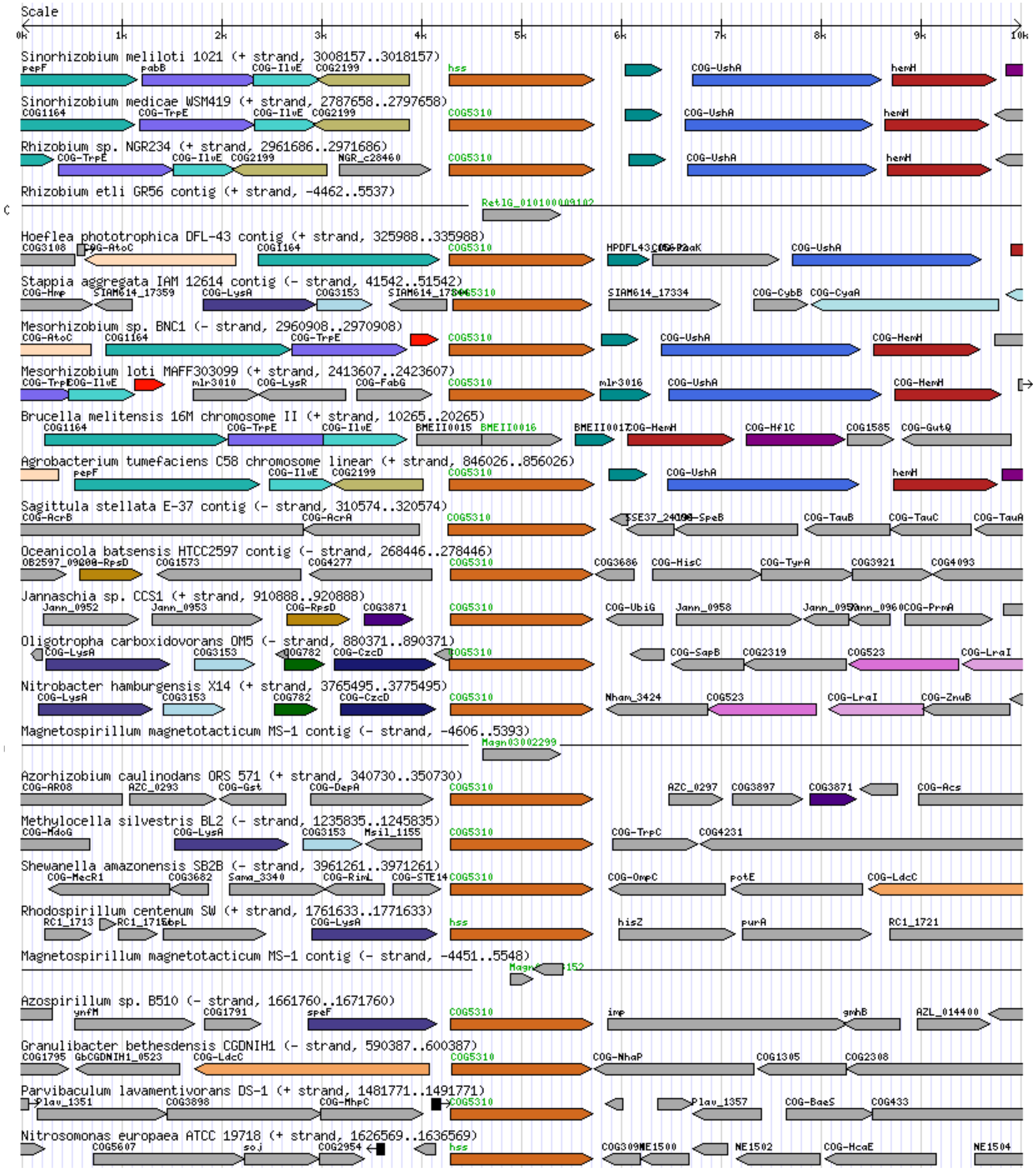

Fig. S2. Genome context of the *hss* gene in selected bacteria.

```
H. pylori      GSHMASMHTVLQIGAGGVGSVVAHKMGMNRDVFKNIILASRSLDKCYAIKESMLKKGLGE 60
S. meliloti    -----MKKNVLIIGAGGVAQVVAHKCAQNSDVLGDIHIASRTVDKCRKIVESVREKKSLK 55
                      :.** ******..***** . * **: :* :***::***  * **: :*   :

H. pylori      ----IGVEQVDADDTQALVALIQKYKPKVVINVALPYQDLTIMQACLETKTHYIDTANYE 116
S. meliloti    TEVKLEAHALDALDVEATKALIVSTGSQIVINVGSAFVNMSVLRACMDTGVAYMDTAIHE 115
                   : .. :** *.:*  *** .   ::****.  : ::::::**::* . *:*** :*

H. pylori      HPDLA----KFEYKEQWAFDRAYKEARILGVLGAGFDPGVTNAYVAHAQRHHFDTIHTLD 172
S. meliloti    EPNKICETPPWYGNYEWKRAAECKEKGITAILGVGFDPGVVNAYARLAKDEYFDKITDVD 175
               .*:       :  : :*      **  * .:**.******.***.  *: .:**.*  :*
H. pylori      ILDCNAGDHKRPFATNFNPEINLREVSSKGRYYENGKWIETKPLEIKQVWAYPQIGEMDS 232
S. meliloti    IVDINAGNHGKYFATNFDPEINFREFTGVVYSWQKGAWQTNRMFEVGKEFDLPVVGKRQA 235
               *:* ***:* : *****:****:**.:.    :::* *  .: :*: : :  * :*: ::

H. pylori      YLLYHEELESLVKNIKGLRRARFFMTFSQNYLTHMKCLENVGMLGIKEIE-HQGVKIVPI 291
S. meliloti    YMTGHDEVHSLSKNMDGA-DVRFWMGFGDHYINVFTVLKNLGLLSEQPVKTAEGLEVVPL 294
               *:  *:*:.** **:.*   .**:* *.::*:. :. *:*:*:*. : ::  :*:::**:

H. pylori      QFLKTLLPDPATLAKDTTGKTNIGCYMTGIKNNQDKTLYIYNVCDHKKCYEEVGSQAISY 351
S. meliloti    KVVKAVLPDPASLAPGYEGKTCIGDFVKGLKDGREREVFIYNVADHRQAYEEVGSQGISY 354
               :.:*::*****:** .  *** ** ::.*:*:.::: ::****.**::.*******.***

H. pylori      TTGVPAMCAAKMICNDTWSADHFRAGVFNIEELNTDPFMEELIKQGLPYEVIER------ 405
S. meliloti    TAGVPPVAAAMLIASGEWDVRQ----MANVEELPPRPFLDILNRIGLPTRIKDEQGDRPL 410
               *:*** :.** :*... *.. :    : *:***   **:: * : *** .: :.

H. pylori      ---405
S. meliloti    SFS413
```

Fig. S3. Clustal Omega protein alignment of the *H. pylori* CASDH and *S. meliloti* Casdh sequences, with key amino acid residues identified in *H. pylori* [17] indicated as follows: Blue: NADP binding residues. Red, dimerization interface residues. Green: residues involved in substrate specificity.

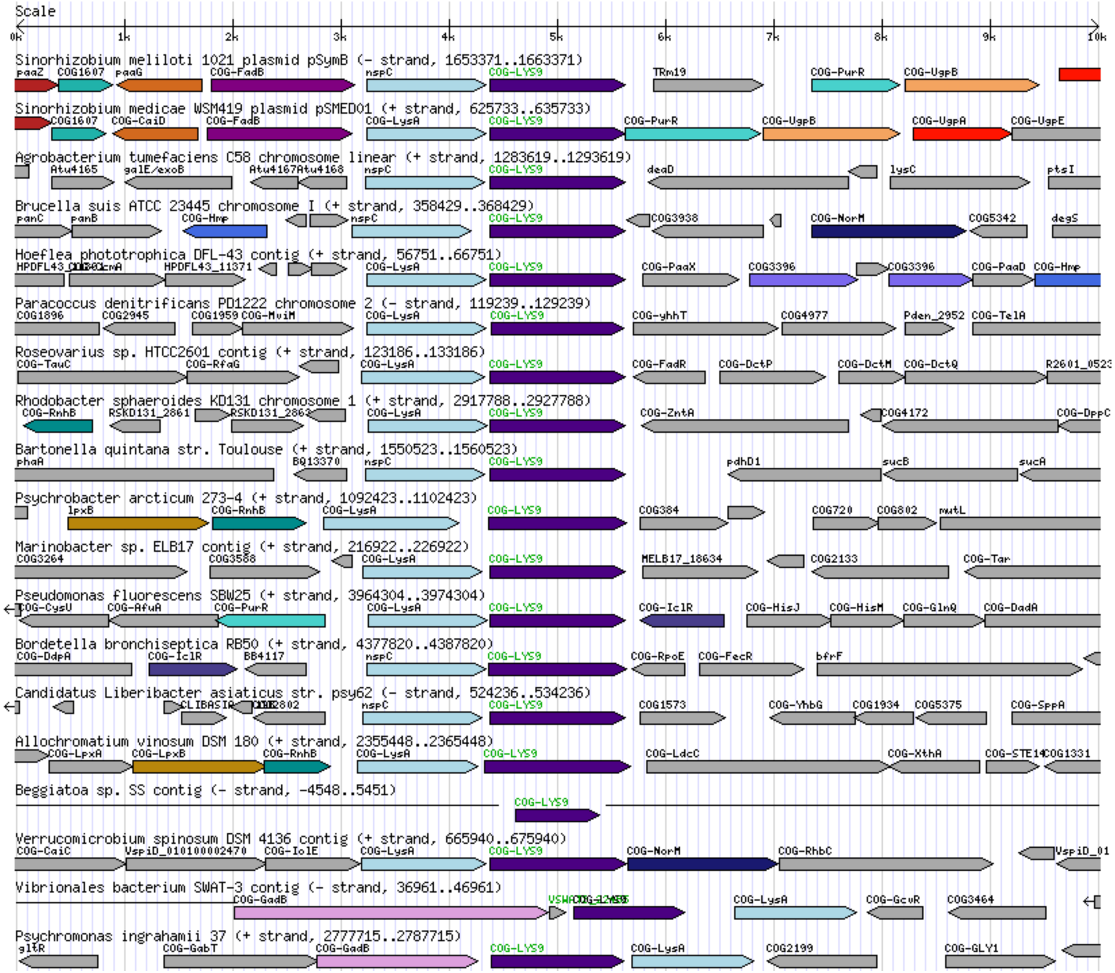

Fig. S4. Genome context of the *casdh* gene in selected bacteria.
